# Determinants of Maternal Insulin Resistance during Pregnancy: An Updated Overview

**DOI:** 10.1155/2019/5320156

**Published:** 2019-11-19

**Authors:** Ulla Kampmann, Sine Knorr, Jens Fuglsang, Per Ovesen

**Affiliations:** ^1^Steno Diabetes Center Aarhus, Aarhus University Hospital, 8200 Aarhus N, Denmark; ^2^Department of Obstetrics and Gynecology, Aarhus University Hospital, 8200 Aarhus N, Denmark

## Abstract

Insulin resistance changes over time during pregnancy, and in the last half of the pregnancy, insulin resistance increases considerably and can become severe, especially in women with gestational diabetes and type 2 diabetes. Numerous factors such as placental hormones, obesity, inactivity, an unhealthy diet, and genetic and epigenetic contributions influence insulin resistance in pregnancy, but the causal mechanisms are complex and still not completely elucidated. In this review, we strive to give an overview of the many components that have been ascribed to contribute to the insulin resistance in pregnancy. Knowledge about the causes and consequences of insulin resistance is of extreme importance in order to establish the best possible treatment during pregnancy as severe insulin resistance can result in metabolic dysfunction in both mother and offspring on a short as well as long-term basis.

## 1. Introduction

The physiology of insulin resistance during pregnancy is fascinating, from an evolutionary point of view designed to limit maternal glucose utilization and thereby shunt an adequate amount of supply to the growing fetus, which requires most of its energy source as glucose. Hence, the degree of maternal insulin resistance established during pregnancy is associated with the degree of glucose flux from the mother to the fetus [1]. Maternal hyperglycemia leads to fetal hyperglycemia and hyperinsulinemia, which cause fetal macrosomia—one of the most common and serious complications of maternal diabetes and obesity. Over the last decades, there has been an increase in obesity among women in the reproductive age, leading to a deterioration in the physiologic insulin resistance with a negative impact on the intrauterine environment, affecting perinatal programming and potentially resulting in metabolic dysfunction in the offspring.

The determinants and causal mechanisms of insulin resistance in pregnancy are complex and still not completely revealed, but in this review, we seek to give an overview of the hormonal and metabolic factors that have been described to have a role in the development of insulin resistance in human pregnancy until now.

## 2. The Development of Insulin Resistance in Pregnancy

**Figure 1:**
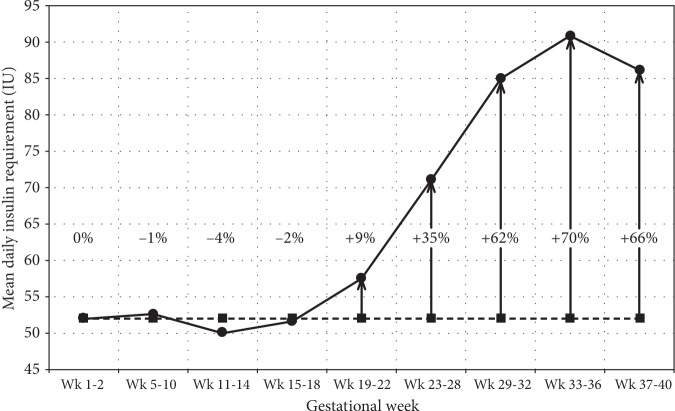
Mean daily insulin requirements at the different time intervals during pregnancy for women with type 1 diabetes. The dashed line represents the prepregnancy insulin requirement, and the numbers in percentages indicate the increase or decrease in percentage relative to prepregnancy levels (Skajaa et al. [4]).

Insulin resistance is the decreased biological response to a given insulin dose, be it endogenous or exogenous, in the target tissue (liver, muscle, or adipose tissue) [2]. In a normal pregnancy, maternal tissues become increasingly insensitive to insulin. A 50-60% decrease in insulin sensitivity is seen with advancing gestation both in women with normal glucose tolerance and in women with gestational diabetes [3]. In women with normal glucose tolerance, the changes in insulin sensitivity are overcome by a sufficient increase in insulin production by pancreatic beta cells, but in women with diabetes, endogenous insulin secretion is insufficient during pregnancy [3]. In women with type 1 diabetes, insulin requirements increase averagely by 70% during pregnancy [4] (Figure 1). From gestational week 11 to week 16, there is a minor decrease in insulin requirements as a consequence of an improvement in insulin sensitivity that is known to increase the risk of especially nocturnal hypoglycaemia in women with type 1 diabetes. From week 20, there is a substantial increase in insulin requirements as a result of a marked decrease in insulin sensitivity until week 33 [4].

The pattern of insulin requirements in pregnancy varies between women with type 1 and type 2 diabetes, suggesting a differential effect of pregnancy-mediated insulin resistance. Women with type 2 diabetes require a much greater increase in insulin dose from the start to the end of each trimester, and insulin requirements do not decrease in early and late pregnancy as is the case in women with type 1 diabetes [5, 6].

The changes in insulin sensitivity through pregnancy are believed to be caused partly by hormones from the placenta and partly by other obesity- and pregnancy-related factors that are not fully understood.

## 3. Molecular Mechanisms

The known changes in glucose metabolism in gestational diabetes are described in detail by Catalano [3]. The metabolic dysfunction includes impaired insulin response in peripheral tissues, decreased hepatic suppression of glucose production during insulin infusion, and decreased insulin stimulated uptake in skeletal muscle. Skeletal muscle and adipose tissue together represent the major sites of glucose disposal in the body. In a normal pregnancy, insulin-mediated whole body glucose disposal decreases by 50% [7]. This is related to a decrease in the postreceptor insulin signaling cascade, specifically a decreased insulin receptor substrate 1 tyrosine phosphorylation, leading to a decreased ability to translocate the glucose transporter GLUT 4 to the surface of the muscle cell, which in turn mediates the transportation of glucose into the muscle cell. [2, 8]. The decrease in insulin receptor tyrosine kinase phosphorylation and receptor tyrosine kinase activity is seen both in pregnant women with normal glucose tolerance and in women with gestational diabetes, but the decrease is reversed postpartum in women with normal glucose tolerance [8], whereas it is not improved significantly in women with gestational diabetes postpartum [3]. The persistent insulin resistance in gestational diabetes may be related to inflammatory factors, mediated by action of placental hormones and other cytokines, affecting the postreceptor insulin signaling cascade [3, 7].

In hyperinsulinemic euglycemic clamp studies with an insulin infusion rate of 1.0 mU/kg/min, combined with stable isotopes of glucose (6,6-^2^H_2_ glucose) to estimate endogenous (primarily hepatic) glucose production, there was a significant 30% increase in hepatic glucose production by late pregnancy in women with normal glucose tolerance and gestational diabetes, but there were no differences between the groups. During the clamp, there was only an 80% suppression of hepatic glucose production in the women with gestational diabetes, compared to the healthy pregnant women, where glucose production was suppressed by 95% [3]. Clamp studies conducted as a dose response study in severely insulin-resistant nonpregnant type 2 diabetic patients have shown that endogenous glucose production is not completely suppressed before a dose as high as 5.0 mU/kg/min is given [9], but it remains to be elucidated to what extent the endogenous glucose production and the insulin signaling cascade are affected during pregnancy in patients with type 2 diabetes and severe insulin resistance.

## 4. Insulin Resistance and Obesity

Obesity is a pivotal cause of insulin resistance, and the changes in insulin sensitivity through pregnancy are partly related to maternal fat mass. Thus, changes in insulin sensitivity in early pregnancy in lean women are inversely related to changes in maternal fat mass, and a significant increase in fat mass in both lean and obese women is seen during pregnancy. [3]. Lipid metabolism is also affected as a consequence of insulin resistance during pregnancy leading to doubled or tripled concentrations of triglycerides and cholesterol late in gestation. The increase in free fatty acids is consequently due to an attenuated effect of insulin on lipolysis [2].

In obese pregnant women, upper-body fat depots are predominant and the predilection for storing fat centrally increases the concentrations of free fatty acids and lipotoxicity that leads to inflammation, endothelial dysfunction, a decrease in trophoblast invasion, and consequently a reduction in placental metabolism and function [10]. The combination of excess lipid and glucose supplies to the fetus and a suboptimal placental function and metabolic environment in utero consequently also increase the risk of metabolic disease in the offspring [10].

As obesity plays a central role in insulin resistance, different aspects of it will also be addressed in further details in the following sections.

## 5. Polycystic Ovary Syndrome (PCOS)

In women with Polycystic Ovary Syndrome (PCOS), obesity is not always present, but visceral adiposity is predominant due to higher testosterone levels in both obese and nonobese women with PCOS. Visceral adiposity amplifies and worsens metabolic and reproductive outcomes and increases insulin resistance through an increase in lipolysis and free fatty acids, leading to compensatory hyperinsulinemia, which in turn increases adipogenesis and inflammatory adipokines and increases insulin resistance even more [11]. Obese women with PCOS have decreased levels of sex hormone-binding globulin (SHBG), increased testosterone, more hirsutism, higher glucose levels, and increased insulin resistance, leading to a higher risk of menstrual irregularity, infertility, miscarriage, hypertension in pregnancy, gestational diabetes, premature delivery, biochemical and clinical hyperandrogenism, glucose-intolerance, type 2 diabetes, and the metabolic syndrome. In addition, the body mass index (BMI) has a greater impact on insulin resistance in women with PCOS in general compared to healthy controls [11]. In pregnancy, PCOS is associated with a higher gestational weight gain and accordingly worsened pregnancy and infant outcomes [12]. Some studies however indicate that nonpregnant lean women with PCOS might be as insulin-sensitive as age- and weight-matched controls, as this has been shown in a few studies using the hyperinsulinemic euglycemic clamp technique. These studies thus underline the importance of being normal weight to avoid insulin resistance [13–15].

## 6. The Placenta

Placenta indeed plays a crucial role in the development of insulin resistance in pregnancy. Thus, it is noteworthy that there is a characteristic rapid restoration of glucose homeostasis immediately after the expulsion of the placenta at delivery, but the potential linkages between the placenta and insulin resistance still remain to be elucidated in further detail.

Placenta is placed as the interface between the maternal and fetal environments, and alterations in placental structure and function may influence fetal growth and development. The exchange of glucose between a mother and a fetus is pivotal for fetal growth and well-being, and glucose is a major placental energy substrate. Due to the important role of the placenta and the glucose metabolism herein, it readily influences the complications of maternal diabetes. In relation to the duality between the maternal glucose homeostasis and placental function, a recent study demonstrated toxic effects of insulin resistance and circulating insulin levels on placental tissues, at least in early pregnancy [16].

Maternal obesity and diabetes are associated with specific structural placental changes such as increased placental weight, increased angiogenesis, and delayed villous maturation [17]. It is suggested that these changes are closely related to the level of glycemic control in pregnancy. In addition, placental function may be compromised in pregnancies complicated by maternal obesity and diabetes. It may also be a result of impaired mitochondrial function because of increased oxidative stress [18]. In addition, the activity of specific amino acid transporter proteins in placenta may be altered [19].

The specific association between placental structure and function and the degree of peripheral insulin resistance remains to be explored, but potential linkages between the placenta and insulin resistance have been suggested to be mediated through the secretion of hormones, cytokines, and adipokines or through the release of other substances from the placenta to the maternal circulation.

## 7. Hormones

During pregnancy, many hormonal axes are influenced by placenta. The placenta secretes pregnancy-specific hormones into the maternal circulation. In other instances, the placenta secretes hormones that circumvent the normal hormonal regulation or even take over normal regulatory pathways. Placental hormones may also influence hormone secretion by structural similarities to hormones also found in the nonpregnant state.

Examples of hormones specific to pregnancy are human chorionic gonadotropin (hCG), human placental lactogen (hPL), and human placental growth hormone (hPGH). Prolactin, estradiol, and cortisol are examples of hormones that are found in increasing amounts in the maternal circulation during pregnancy.

An example of the take-over of the maternal metabolism is the growth hormone axis: pituitary growth hormone (GH) is gradually replaced by hPGH during pregnancy. It almost completely replaces pituitary growth hormone in the maternal circulation by approximately 20 weeks of pregnancy and is secreted tonically rather than in a pulsatile fashion, unlike GH [20]. Furthermore, the serum level of hPGH is comparable to acromegalic levels, i.e., 10 times higher than GH outside pregnancy. Especially, hPGH has been described as a somatogenic rather than a lactogenic bioactivity compared to GH [21]. The hPGH may also have the same diabetogenic effects as pituitary growth hormone such as hyperinsulinemia, decreased insulin-stimulated glucose uptake and glycogen synthesis, and impairment of the ability of insulin to suppress hepatic gluconeogenesis. Some of these effects have been demonstrated in rodents *in vitro* [22], whereas the effects during human pregnancy are less evident [23]. It appears that hPGH is the driver of the levels of insulin-like growth factors (IGFs) during pregnancy [24, 25], thereby indicating an interplay between GH, hPGH, IGFs, and insulin-like growth factors binding proteins (IGF-BPs), which might be the pathway coupling insulin resistance and the growth hormone axis during pregnancy. Interestingly, the pregnancy-associated plasma protein A (PAPP-A), which due to its first trimester abundancy is used for first trimester risk assessment at the time of the nuchal translucency scan, appears to be intricately associated to the IGF-BPs and thereby the growth hormone-IGF axis during pregnancy [26]. Such findings clearly implicate a role for the growth hormone-IGF axis during pregnancy.

Estradiol, progesterone, prolactin, cortisol, hPL, and hPGH have previously been described to be mediators of the change in insulin sensitivity during gestation. However, in a study by Kirwan et al., correlations were performed on the changes in plasma levels of cortisol, leptin, human chorionic gonadotropin (HCG), estradiol, progesterone, and hPL compared with the changes in insulin sensitivity during pregnancy. The authors only found a significant correlation between insulin sensitivity and cortisol levels [27]. McIntyre et al. [23] found that, instead, IGFBP1, triglycerides, and leptin correlated significantly with estimates of maternal insulin sensitivity. Thus, at present, no single hormone has been found to explain the insulin resistance of pregnancy.

Many placental hormones have very short half-lives in the maternal circulation, and within 24 to 48 hours after delivery, the effect of such placental hormones have vanished and nonpregnant physiology is in many ways restored [28, 29]. A clinical implication of this is that within one or two days after delivery, restoration of insulin requirements towards prepregnancy levels, or even lower, is seen in mothers with type 1 diabetes [30].

## 8. Cytokines and Adipokines

Alterations in cytokines have also been studied as a potential pathophysiological mechanism behind the increase in insulin resistance in pregnancy. White adipose tissue and the placenta act as endocrine organs, secreting adipokines and cytokines such as leptin, adiponectin, tumor necrosis factor alpha (TNF-*α*) and interleukin-6 (IL-6) [3]. Nayak et al. investigated changes in cytokine levels (IFN-*γ*, IP-10, IL-1*α*, MIP1-*α*, adiponectin, and leptin) and ICAM1 (a cell surface glycoprotein expressed on endothelial cells and cells of the immune system that can be measured due to shedding) in overweight and obese women during pregnancy and found that IL-1*α*, ICAM1, and adiponectin were inversely associated with insulin and insulin resistance, but the authors concluded that further studies are required to confirm the role of these cytokines in glucose and insulin metabolism in obese pregnant women [31]. TNF-*α* has also been found to correlate inversely with insulin sensitivity in pregnant women with normal glucose tolerance and in women with gestational diabetes [27]. It has been shown that TNF-*α* contributes to insulin resistance through impairment of insulin signaling by increasing serine phosphorylation of insulin receptor substrate- (IRS-) 1 and diminishing insulin receptor (IR) tyrosine kinase activity [7]. Thus, TNF-*α* may be produced in the placenta and skeletal muscle to induce or exacerbate insulin resistance. However, in a later study by McIntyre et al., an association between TNF-*α* and insulin resistance in healthy pregnant women could not be confirmed [23], whereas Catalano supports that TNF-*α* plays a significant role in the development of insulin resistance in women with gestational diabetes [3].

All the available studies have only examined the inflammatory changes in pregnant women with normal glucose metabolism or gestational diabetes. Studies on how inflammatory markers and hormones affect insulin sensitivity in pregnant women with type 1 diabetes, type 2 diabetes, and severe insulin resistance remain to be performed.

## 9. Exosomes

As circulating levels of placental hormones do not correlate well with maternal insulin sensitivity [27], other, previously unrecognized, mechanisms may be involved. New data suggest that exosomes (i.e., membrane-derived nanovesicles) may play a role throughout gestation, including mediation of a placental response to hyperglycemia and insulin sensitivity. Exosomes are secreted from both the placenta and adipose tissue [32] and have been demonstrated to contain many different substances that are also found intracellularly in their tissue of origin. Such substances may relate to immunomodulatory processes in placental exosomes, potentially linking inflammatory processes and insulin resistance. The content in exosomes derived from adipose tissue—e.g., leptin and adiponectin—links readily insulin resistance to the secretion of exosomes [32]. It has been found that the levels of circulating exosomes (total and placenta-derived) are higher in gestational diabetes compared to normal pregnancies across gestation [33] and hyperglycemia increases the release of exosomes from primary human first trimester trophoblast cells [34], suggesting an association between the circulating levels of placental exosomes and the maternal metabolic status during pregnancy.

## 10. Physical Activity

It is well established that exercise or physical activity reduces insulin resistance in nonpregnant human beings by stimulating the glucose transporters onto the surface of skeletal muscle cells and thereby improving glucose uptake [35, 36]. Furthermore, the level of exercise has been associated with a decreased risk of type 2 diabetes for decades [37]. A recent systematic review and meta-analysis also demonstrated a reduction in blood glucose concentrations in women with and without diabetes in pregnancy both during and following acute as well as chronic exercise interventions [38]. In addition, several studies have shown that exercise can delay or prevent the occurrence of gestational diabetes mellitus [39], not to mention the beneficial effects of exercise in the treatment of women with gestational diabetes [40]. Thus, in a recent randomized controlled trial (RCT) study, exercise initiated early in pregnancy, lasting at least 30 minutes 3 times per week, reduced the risk of gestational diabetes significantly in overweight and obese pregnant women by 45.8% [41]. van Poppel et al. have also shown that in a group of overweight and obese pregnant women, moderate-to-vigorous physical activity was associated with an improved insulin sensitivity and insulin response at 32 weeks of pregnancy [42].

## 11. The Microbiome

One of the factors that might also have an important impact on glucose homeostasis is the gut microbiota. Several studies have shown differential microbial abundance between healthy individuals and individuals with prediabetes, insulin resistance, and type 2 diabetes [43–45]. Fecal transplantation from lean to obese individuals with the metabolic syndrome has shown an improvement in the recipient's peripheral insulin sensitivity, and the gut microbial diversity also increased after transplantation [46].

Koren et al. demonstrated a significant change in the gut microbiota of 91 pregnant women with loss of bacterial richness and an increase in the beta-diversity from the first to the third trimesters. In the same study, germ-free nonpregnant mice were inoculated with stool samples from the study cohort and it was found that the third trimester microbiota induced greater adiposity and insulin resistance compared with first trimester stool inoculation [47]. These results indicate that in pregnancy, the gut microbiota may contribute to the maternal metabolic changes. Likewise, a Danish study investigated gut microbiota profiles in 50 women with gestational diabetes and in 157 healthy pregnant women and found that in the third trimester of pregnancy, gestational diabetes was associated with an altered gut microbiota compared to pregnant women with a normal glucose tolerance [48]. Also, a Finish group have previously shown a reduction in the prevalence of gestational diabetes from 36% to 13% in lean pregnant women treated with probiotics [49]. However, in a newly published RCT where 439 overweight or obese pregnant women were treated to evaluate whether the risk of gestational diabetes and/or glucose metabolism may be improved by fish oil and/or probiotic supplements, no beneficial effect on neither the risk of gestational diabetes nor glucose metabolism measured as fasting glucose, insulin, or insulin resistance (HOMA) was found [50]. A possible explanation for the difference in the two studies could be that the metabolic burden of obesity in the second study was so severe that it could not be overcome by the potentially beneficial effects of fish oil or probiotic in regulating glucose metabolism.

On the other hand, two recent meta-analyses have shown that the use of probiotics was associated with an improved glucose and lipid metabolism in pregnant women and might also reduce the risk of gestational diabetes [51, 52]. Yet another meta-analysis showed that supplementation with probiotic reduced insulin resistance (HOMA-IR) and fasting serum insulin in women with gestational diabetes significantly, compared to healthy pregnant controls [53].

The question whether gut modification could be an effective tool in reducing insulin resistance in pregnant women is complicated, and studies are ongoing. Results differ as the human gut houses a complex microbial ecosystem, and the present studies have used different either pre- or probiotics or multistrain probiotics, making it difficult to compare studies and to make a final conclusion at the moment.

## 12. Genetic Predisposition and Later Life Insulin Resistance

The genetic heritage of a woman can predispose her to excess insulin resistance, but early life experiences can also alter insulin resistance later in life.

The genetic heritability of insulin resistance in pregnancy depends on a woman's genetic heritability for obesity, PCOS, gestational diabetes, and/or type 2 diabetes, conditions that all contribute to insulin resistance and for most share at least some genetic composition.

Genome-wide association studies have described more than 250 loci being associated with type 2 diabetes. A minor part of these loci is associated with a higher susceptibility for increased insulin resistance; however, most loci are associated with altered insulin secretion and beta-cell function [54]. The genetic composition of type 2 diabetes is dominated by common alleles with small impact on the risk of disease [54]. No genome-wide association studies (GWAS) solely describing insulin resistance in pregnancy exist. However, a GWAS study performed in participants from the HAPO (Hyperglycemia and Adverse Pregnancy Outcome) study found genes and SNPs previously described to be associated with altered glycemic traits in nonpregnant populations as also being associated with an altered glycemic response in the pregnant women [55]. The study also described two novel associations between altered glucose response in pregnancy and two genetic loci (HKDC1 and BACE2) [56]. Another GWAS study from 2012 describes how genetic variants in CDKAL1 and near MTNR1B are associated with gestational diabetes in Korean women [56]. The genetic variants found in the Korean study, along with other common type 2 diabetes susceptibility polymorphisms, have been described to be associated with gestational diabetes in candidate gene studies [57]. Also, the G7G genotype of TNF-alpha 308 G/A polymorphism increases insulin levels and insulin resistance among women with gestational diabetes [58]. In nondiabetic pregnant women, genetic variants of KCNJ11 and MTNR1B are associated with insulin resistance during pregnancy [59]. Focusing on inflammatory processes during pregnancy, researchers from the HAPO study demonstrated how variants in six out of 31 inflammatory pathway genes were associated with maternal metabolic traits using a candidate gene approach [60].

As with type 2 diabetes, GWAS studies have identified a number of common variant loci associated with obesity. More than 500 loci have been identified, but the loci combined only explain approximately 4% of the variation in BMI [61].

## 13. The Influence of Early Life Environment on Later Life Insulin Resistance

No studies on early life experiences and the development of insulin resistance in pregnancy exist, but epidemiological and clinical studies have illustrated how an early life environment has the ability to influence later life health. Both shortage and excess in intrauterine nutrition alter the risk of impaired glucose tolerance, type 2 diabetes, cardiovascular disease, and obesity in adolescent and adult life.

In 1998, a landmark study, with findings from the Dutch famine or “Hunger Winter” during World War II, was published. The follow-up study of subjects exposed to the famine in utero showed elevated 2-hour blood glucose during an oral glucose tolerance test (OGTT) compared to nonfamine exposed controls, especially if the famine exposure occurred in the second or third trimester [62]. The intrauterine exposure can be less dramatic than famine; hence, being born with low birthweight also increases the risk of later life metabolic disease [63]. Likewise, studies have reported a causal link between maternal smoking and subsequent increased risk of insulin resistance, type 2 diabetes, and hypertension, although the evidence here is weaker than that for overweight/obesity [64]. At the other end of the spectrum, intrauterine exposure to hyperglycemia and/or maternal obesity also increases the risk of later life obesity and impaired glucose tolerance [22, 65, 66]. A recent study describes how intrauterine exposure to hyperglycemia affects the offspring in a sex-specific way, with females being more prone to the effects [67]. The same study describes how intrauterine exposure to hyperglycemia to a greater extent impairs OGTT-derived indexes of insulin resistance compared to indexes of insulin secretion. Maternal insulin resistance results in a surplus of glucose and lipids, leading to overnutrition of the fetus and consequently an increased risk of metabolic disease later in life [22]. Thus, it could be hypothesized that insulin resistance in pregnancy is also influenced by early life exposure.

## 14. Mechanisms Linking Early Life Exposure to Later Life Health

Early life exposure affects not only the woman's own risk of increased insulin resistance in pregnancy but also her children's risk of disease later in life. However, the pathophysiological mechanisms behind the changes observed in epidemiological and clinical studies are complex and unclear.

The direct effects of hyperglycemia and hyperinsulinemia on the adipose tissue, muscle, liver, blood vessels, and pancreas are possible pathogenic pathways. Several studies using combinations of hyper/euglycaemic and hyper/euinsulinemic clamps have reported altered gene expression in fat and adipose tissue during acute or short-term exposure to hyperglycemia [68, 69].

However, changes in the DNA sequence cannot explain the alterations in metabolic phenotype observed from generation to generation. Epigenetic modifications have therefore been proposed as a possible mechanism by which early life exposures induce long-term effects. Epigenetic modifications include DNA methylation, histone modifications, chromatin-modifying proteins, and altered regulation or expression of noncoding RNA. Histone modifications include acetylation of lysines, methylation of lysines and arginines, and phosphorylation of serines and threonines. Histone modifications can alter the recruitment and binding of DNA-regulatory proteins and hereby change DNA replication and transcription. Noncoding RNA are involved in gene silencing, X-inactivation, genomic imprinting, and germ cell reprogramming, all involving epigenetic modifications. Methylation of cytosine is the best-studied epigenetic mark and entails the addition of a methyl group to the C5 position of the cytosine ring. The methylated cytosine is usually followed by guanines and thus called CpG islands. This modified DNA sequence is distributed over the majority of the human genome and is often involved in transcription repression [70]. Early life modifications to these epigenetic marks, especially in utero, create an epigenetic memory and program the offspring's later life phenotype. Epigenetic changes have been observed as a consequence of both intrauterine exposure to famine and maternal gestational diabetes and type 2 diabetes, thereby creating a pathophysiological link between early life exposure and later life metabolic disease [71–73].

## 15. Conclusion

Insulin resistance during pregnancy is accentuated in situations with diabetes, obesity, and inactivity and can become a serious condition with important implications for pregnancy outcome and long-term morbidity for the mother and offspring. The mechanisms lying behind the insulin resistance of pregnancy are multifaceted and probably involve both hormonal, placental, genetic, and epigenetic contributions and modifications from the level of activity, diet/microbiome, and overweight/obesity.

Knowledge about the causes and consequences of insulin resistance in pregnancy is of extreme importance, and it is crucial to strive for a more detailed insight into the mechanisms behind the insulin resistance developing during pregnancy and the impact on the offspring, in order to tailor the best possible treatment for pregnant women with diabetes, beneficial for both the mother and the future generation.
